# Supplementary material for: Identification of human progenitors of exhausted CD8+ T cells associated with elevated IFN-γ response in early phase of viral infection
Source: Nat Commun. 2022 Dec 7;13:7543. doi: 10.1038/s41467-022-35281-7 (PMC9729230; doi:10.1038/s41467-022-35281-7)
Supplement: Supplementary file 1 — Supplementary Information [file 41467_2022_35281_MOESM1_ESM.pdf]

SUPPLEMENTARY NOTE 1

Analysis of viral sequencing

Viral genomes were extracted from plasma and sequenced as described previously [1]. Viral genomes for four individuals (CH-3023, CH-3240, CL-360, CL-MCRL) were sequenced and reported previously [2]. Both consensus (Sanger) and deep sequencing were performed for 11 individuals, and in 3 individuals only consensus or partial genome sequences were available (Supplementary Table 2). T/F viruses were identified as previously described [3]. Briefly, haplotype reconstruction was performed from the earliest available sample time-point from samples with 454 or Illumina sequencing to identify all peripheral circulating variants. Phylogenies were constructed containing all variants and for those with star-like structures, the central variant was identified as the T/F virus. PoissonFitter was applied to describe mutations arising from a single T/F virus under no selective pressure by the host immune response. For individuals with only Sanger sequencing, the consensus sequence at the earliest sample was identified as the T/F virus. Viral diversity was calculated using Shannon entropy and utilising in house scripts as previously described [4, 5].

Epitope selection

The set of HLA-restricted peptide epitopes tested in ELISpot assays for each individual were determined from the circulating virus at the earliest viraemic timepoint. For each individual, this subset was selected from three major sets of epitopes. (i) set 1 consisted of autologous T/F virus or earliest viral consensus sequence HLA class I-restricted epitopes (9 to 11 amino acids) that were at least 90% homologous to previous experimentally confirmed epitopes from the Immune Epitope Data Base (IEDB [www.iedb.org]). If >1 T/F was present, then a selection was taken on epitopes that were predicted from the most dominant T/F (with the second virus having a frequency of occurrence <20%); (ii) set 2 consisted of epitopes identified by IEDB epitope prediction tools (using the IEDB-recommended procedure for 9 to 11 amino acids) from the earliest dominant viral sequence with a high predicted binding score (defined as a half-maximal inhibitory concentration [IC_{50}] of 500 nmol), and 90% homologous to experimentally-confirmed epitopes available in the IEDB database; iii) set 3 consisted of potential immune escape variants identified from the longitudinal set of sequences associated with a nonsynonymous substitution that subsequently reached fixation over the course of the infection and with an increased IC50 of 500 nmol. As the total number of potential epitopes to be tested across these three sets was typically large (~200-800 epitopes), a prioritised selection of up to 100 epitopes was undertaken for each individual to account for the total PBMCs available for the ELISpot matrix testing approach and subsequent flow cytometric analyses (see below). A *post hoc* decision process was also undertaken in cases where the number of planned epitopes could not be tested due to limitations in the availability of viable PBMC following the thawing of frozen cells. In these cases, a subset of epitopes from the selected epitope pool was chosen by random sampling across the three pools. Supplementary Table 2 outlines the number of epitopes tested for each individual.

Mathematical model to estimate the rate of immune escape

Viral epitopes were tested longitudinally and mutations occurring within the epitope regions were analysed using in-house scripts, which identified the mutations and their frequency of occurrence within the population. This tool takes in bam files generated from deep sequencing data, the reference sequence, the starting codon position and beginning and end position of the epitope region in nucleotide positioning based on the reference sequence as inputs. The escape variant was defined as any observed mutations away from the wild-type sequence occurring within the epitope. The frequency of the escape variant at each individual sample timepoint was estimated as the sum of the frequencies of each epitope variants carrying one or more mutations.

These longitudinal frequencies were then used to estimate the rate for $CD8^+$ T-cell epitope escape by fitting the data to a population dynamics model that describes the dynamics of viral variants under the presence of cytotoxic T cell responses [6]. The model predicts that the frequency of the escape variant *f(t)* is:

$$f(t) = \frac{f_0}{f_0 + (1 - f_0)e^{-kt}} \qquad (1)$$

where k *is* the rate of escape. There is an assumption in this model that the escape variant is present during the initial phase of time (t) at zero, and its frequency is given by f_0. In some cases where the escape variant was not observed in the earlier time points, an estimate of $1/(n+1)$ replaces a frequency of 0, where n is the average coverage of the corresponding time point from the deep sequencing data. This estimation was carried out in R using non-linear least-squares approach for non-linear models. The time needed for the escape variant to achieve 50% of the circulating viral population is obtained from the following formula:

$$T_{50} = \frac{\log\left(\frac{1}{f0} - 1\right)}{k}$$

IFN-γ ELISpot assay

ELISpot assays for IFN-γ production were performed as previously described [7]. Briefly, samples were screened for responses in a matrix format using pools with ≤5 peptides. For each sample, groups of 150,000 PBMC were incubated overnight with peptides, a positive control, anti-CD3 antibody (Mabtech, Sweden), and three negative control wells with media only. Responses with greater than 20 SFU/million PBMC were considered positive. Positive responses were confirmed by stimulation with a single peptide and 200,000 PBMC. Positive responses were defined as exceeding the background level, defined as the mean plus three times the standard deviation of SFU in the negative control wells.

Flow cytometry

Peripheral blood mononuclear cells (PBMCs) were thawed in RPMI and washed with PBS containing 1% BSA. Cells were stained with PE-conjugated HCV-specific class I dextramers (Immudex, Copenhagen, Denmark) at room temperature, followed by viability staining (LIVE/DEAD™ fixable blue for analysis or fixable yellow for sorting) (Invitrogen, Carlsbad, CA) and staining with panels of surface or intracellular antibodies detailed below.

Flow cytometry was performed using the LSR Fortessa analyser, and FACSAria III and Influx sorters (BD Biosciences, San Diego, CA). Flow cytometry data was analysed using FlowJo software version 10.4.2 (BD Biosciences, San Diego, CA). Gates for CD127, PD-1, CD38, and Tim-3 were determined with fluorescence minus one (FMO) controls.

Two panels of antibodies were used for flow cytometry analysis (listed in Supplementary Table 7). Both panels contained CD4-FITC, CD19-PE-Cy5, CD3-APC-Cy7, CD8-AF700, PD-1-BV510. The first panel included: TIM-3-PerCP-Cy5.5, TIM-3-BV421, CD38-BV605, CD127-BV650, 2B4-PE-Vio770, CD160-AF647 and CD127-BV650. Dextramers (Immudex) were conjugated with PE. For KLRG1 staining, cells were incubated with primary antibody Biotin anti-KLRG1 followed by incubation with secondary antibody PE-CF594 Streptavidin. The second panel included CCR7- BV421, CD45RO-PE-Cy-7, CD27-BUV395 and CTLA-4-PE-CF594. For the intracellular staining, cells were fixed and permeabilized using fix/perm buffer from transcription factor buffer set kit (BD Biosciences) at 4°C for 35 minutes and stained with against intracellular markers T-bet-BV711 and EOMES-eFluor660 at 4°C for 30 minutes. The cells were then washed twice with Perm/Wash buffer (BD Biosciences) and fixed with PBS containing 1% paraformaldehyde.

A separate panel was used for flow cytometry for sorting and single-cell RNA-sequencing including: CCR7-BV421, CD3-BV480, CD122-BV650, CD95-BV786, CD38-APC, CD8-APC-R700, PD-1-PE-CF594, CD19-PE-Cy5, CD127-PE-Cy7, CD45RA-FITC, KLRG1-PerCP-Cy5.5 and CXCR3-APC. Dextramers (Immudex) were conjugated with PE. For markers with non-discrete expression profiles such as CD127, PD-1, CD38 and Tim-3, positivity was determined by fluorescent-minus-one (FMO) controls. The mean fluorescence intensity (MFI) values obtained from the index sorting were used to identify protein expression values of individual cells and to validate scRNA-seq gene expression profiles.

Statistical analysis

Statistical analyses were performed using GraphPad Prism 7.0 (GraphPad Software, Inc., La Jolla, USA) and R [8]. Data was expressed as the median with interquartile range and analysed using non-parametric statistics. Analysis of ELISpot data was performed assuming positive responses only with values above

30 SFU/million PBMC. Epitope-specific immune responses with a single value <30 SFU were excluded from downstream analysis. For flow cytometric data, comparisons of gated populations between infection outcome and between time windows were performed using Wilcox sum-rank tests, and p-values less than 0.05 were considered significant. Regression and correlation analyses were performed in R using function "lm" and "ggplots2" libraries. Regression analysis between magnitude of IFN-γ production and rate of escape, the maximum value of the IFN-γ ELISpot measured for each epitope within the first 16 weeks post-infections was considered.

ATAC-seq

ATAC-seq was performed using samples from four individuals at the following timepoints: CH-HOKD 96 DPI, CL-MCRL 115 DPI, CH-3023 73 DPI, and CH-THDS 85 DPI. All timepoints were within the initial 120 DPI and up to 10000 cells were sorted from a combination of dextramer-positive $CD8^+$, dextramer-negative $CD8^+$, total effector $CD38^+CD8^+$, and total effector memory $CD8^+CCR7^-CD45RA^-$ populations. For HCV-specific (dextramer) responses in individual CH-THDS (KLV-specific) two samples were utilised, and only one for the remaining responses. All HCV-negative responses were in duplicates or triplicates. A total of 16 libraries were generated. DNA library preparations were carried out using a previously published protocol [9]. Briefly, target populations were sorted by flow cytometry (FACSAria III). Cells were then lysed and fragmented in a single reaction (12.5 µl 2X Illumina TDE buffer, 2.5 µl 1% Tween-20, 2.5 µl 0.2% Digitonin, 5 µl water, 2.5 µl Illumina TDE1 enzyme). Samples were incubated at 37°C for 60 minutes and purified using the Zymo DNA Clean and Concentrator-5 Kit (Zymo). Fragments were amplified according to the previously published protocol. The total number of cycles was selected to be the number of cycles required to reach one quarter of maximum qPCR fluorescence. Products were purified using Ampure XP beads (1.5X) and quality controlled using a TapeStation (Agilent Technologies). Libraries were sequenced by paired end (2x75 bp) to a depth of 25 million reads per sample (Illumina Miseq v3).

Sequenced reads were trimmed with Trim_Galore (version 0.4.5_dev) and aligned to GRCh38.p12 using BWA-MEM v0.7.17 [9] in paired-end mode with default parameters. The resulting bam files were deduplicated with MarkDuplicates (Picard) v2.19.0. Prior to further analysis, sample quality was verified using the ATACseqQC package v1.10.1 [10]. Samples were normalised using the normOffsets() function, peak regions across samples defined by mergeWindows() with tol=1000L and max.width = 5000L, and regions annotated by detailRanges() from the Bioconductor package csaw v1.20.0 [11], with default values used unless otherwise specified.

scRNA-seq library preparation

Index sorted cells were collected into 96-well plates. Single-cell RNA-seq libraries were generated with

a modified SmartSeq2 protocol[1]. Briefly, a modified protocol from Picelli et al [12] was used to reduce volumes and concentrations of reagents. Sequencing of the libraries was performed on the Illumina Next-Seq or Mi-Seq machines with high throughput kit 150bp and 250 bp respectively.

scRNA-seq bioinformatic analysis

The overall bioinformatics pipeline for the analysis of the scRNA-seq data was conducted mostly as previously reported by our group [13] with some modifications as outlined below.

Sequence Analysis

Raw sequencing reads were trimmed using Trimmomatic (v0.39) [14] and aligned to the reference genome GRCh38.p13 using STAR (v2.7.3a) [15]. Gene expression was quantified using RSEM (v1.3.1) [3] and Ensembl gene annotation release 99. Scaled transcripts per million (scaled TPMs – TPMs scaled so that the total count per cell is equal to the total aligned read count) were extracted from the RSEM results and used for further analysis.

Quality Control

Downstream analysis was performed in R using packages downloaded from Bioconductor 3.10. Cells were removed from each batch if they did not meet these criteria: less than 30% reads aligned to mitochondrial genes, total reads more than 30,000 and less than the 98% quantile for the batch, number of detected genes more than 400 and less than the 98% quantile for the batch, and number of detected genes less than the 25% quantile for detected genes in mini-bulk controls samples (of 30-50 cells) for the batch. The bulk samples were also removed at this stage.

To assess the factors (sampling time point, individual, epitope, library size) contributing to the transcriptional variance between cells the function *getVarianceExplained* and *plotExplanatoryVariables* functions were used from the scater package in R. Genes expressed in less than 1% of cells were removed from the dataset.

Normalisation and Batch correction

Normalization using the *SCTransform* function from the Seurat package (v4)[16] was applied to the quality-controlled samples, separately for each batch. Feature selection and integration pre-processing was then performed using the *SelectIntegrationFeatures* and *PrepSCTIntegration* functions, after which the normalized data from each batch were integrated together using the *FindIntegrationAnchors* and *IntegrateData* functions in Seurat to form an integrated matrix, used for downstream clustering and dimension reduction steps.

Clustering, differential gene expression and gene signature scores

Dimensionality reduction and clustering was performed in Seurat using the integrated matrix. Principle components analysis (PCA) was performed on the VST normalised data using the 3,000 most variable genes. The first 30 principal components were selected as the most significant based on an elbow plot and used as input for UMAP and clustering. Clustering was performed using the shared nearest neighbour (SNN) modularity optimization-based clustering algorithm (*FindClusters*(resolution = 1, algorithm = 'louvain')) as implemented in Seurat, to obtain 13 clusters. Analysis of reference-based clustering was performed using SingleR (v1.0.6) [17].

Differential gene expression for hypothesis testing was performed using the *FindMarkers* function from the Seurat package to obtain the cluster genes. Notably, the test used was MAST, and batch was used as a latent variable with a log-fold-change threshold of 0.3. Other parameters were kept as default. Signature scores were computed from the single cell transcriptomic matrix as the average log(TPM+1) of all genes in the signature. Differential expression output across all the analyses is reported in Supplementary data 4.

Reference-based annotation of T cell subsets gene signatures with published references of T cell subsets from mouse[18] and human T cell data[19] was performed with SingleR (v1.4.1) with default parameters except for "quantile = 0.8", and using log-transformed gene counts [17].

Gene set enrichment analysis (GSEA)

Gene set enrichment analysis was performed using the R package fsgea (v1.16.0). Normalised enrichment scores (NES) were assessed using the fgsea(…, maxSize = 500, nperm = 10000) function across the curated Molecular Signatures Database (MSigDB) Hallmark, C2 curated gene sets comprising REACTOME, KEGG and Canonical gene sets PID), C5 Gene ontology, and C7 Immunological signature. Customised gene signatures for T cell phenotypes are reported in Supplementary data 5, which were prepared by manually curating published data [19, 20] .

Trajectory inference

Slingshot was applied to the UMAP formed from the integrated gene expression matrices using the getLineages and getCurves functions in the slingshot package [21], and by manually assigning an initial root value. PAGA analysis was performed through SCANPY (v1.7.1) with parameters as recommended [22]. Integrated data matrix obtained from SCTransform normalization method in Seurat v4 was used for initial pre-processing steps and visualization using sc.pp.neighbors and a coarse-grained and simplified graph using sc.tl.paga(). Clusters were calculated using sc.tl.louvain() and visualization was performed using sc.pl.paga() and sc.pl.draw_graph(). Psuedotime analysis was performed using the diffusion map

algorithm (sc.tl.dpt) by manually assigning an initial iroot value. Differential gene expression analysis to identify early- and late-stage of each trajectory was based on grouping cells based on the pseudotime threshold value of 0.5.

Scaled pseudotime were used with Loess smoothing and were calculated as uniformly distributed mapping of the diffusion pseudotime values to preserve the cell order and account for heterogeneous distribution of gaps between pseudotime values. The growth rate or velocity with which T cell phenotypic subsets (T_{EX}, T_{PINT}, T_{M} T_{ML} T_{EFF}) change along the inferred pseudotime trajectories were calculated as the ratio between the difference in cell numbers and the scaled pseudotime values over a window of size 0.05. These values were then plotted using the *geom_smooth()* R function with default parameters.

TCR repertoire analysis

TCR full length alpha and beta sequences were obtained from scRNA-seq data using the software tool VDJPuzzle[1]. In two individuals, single cell TCR Sanger sequencing were also generated, utilising established protocol [23]. For individual CH-3023 Sanger sequencing data were generated for the same two epitope-specific responses with available scRNA-seq, at 75, 101 (additional timepoints), and 196 DPI (existing timepoint). For individual CH-240, all TCRs were generated using only Sanger sequencing from two epitope-specific responses at 71, 99, and 140 DPI (additional timepoints) (see Supplementary data 3). For scRNA-seq data, the α and β chains with the highest expression were used where multiple chains were reported in a single cell. Clones were defined as cells with identical TCR CDR3 amino acid sequences in both α and β chains. Shannon entropy (SE) was calculated using the *entropy* function (with parameter ".base = exp(1)") in the Immunarch R package (v0.6.5), utilising the CDR3 amino acid sequences of both α and β chains. Shannon evenness (SEv) was calculated as SE/log(N) where N is the number of unique sequences used to calculate SE.

Supplementary References

1. Bull, R.A. *et al.* A method for near full-length amplification and sequencing for six hepatitis C virus genotypes. *BMC Genomics* **17**, 247 (2016).

2. Bull, R.A. *et al.* Transmitted/Founder Viruses Rapidly Escape from CD8+ T Cell Responses in Acute Hepatitis C Virus Infection. *J Virol* **89**, 5478-5490 (2015).

3. Bull, R.A. *et al.* Sequential bottlenecks drive viral evolution in early acute hepatitis C virus infection. *PLoS Pathog* **7**, e1002243 (2011).

4. Leung, P., Bull, R., Lloyd, A. & Luciani, F. A bioinformatics pipeline for the analyses of viral escape dynamics and host immune responses during an infection. *Biomed Res Int* **2014**, 264519 (2014).

5. Walker, M.R. *et al.* Clearance of hepatitis C virus is associated with early and potent but narrowly-directed, Envelope-specific antibodies. *Sci Rep* **9**, 13300 (2019).

6. Asquith, B., Edwards, C.T., Lipsitch, M. & McLean, A.R. Inefficient cytotoxic T lymphocyte-mediated killing of HIV-1-infected cells in vivo. *PLoS Biol* **4**, e90 (2006).

7. Bretana, N.A. *et al.* Transmission of Hepatitis C Virus among Prisoners, Australia, 2005-2012. *Emerg Infect Dis* **21**, 765-774 (2015).

8. R Project for Statistical Computing., R Foundation for Statistical Computing., Technische Universität Wien. Institut für Statistik Wahrscheinlichkeitstheorie und Versicherungsmathematik., Technische Universität Wien. Institut für Statistik und Wahrscheinlichkeitstheorie. & University of Washington. Department of Biostatistics. R Project. Wien: Technische Universität Wien, Institut für Statistik, Wahrscheinlichkeitstheorie, und Versicherungsmathematik; 2001. p. 1 online resource.

9. Buenrostro, J.D., Giresi, P.G., Zaba, L.C., Chang, H.Y. & Greenleaf, W.J. Transposition of native chromatin for fast and sensitive epigenomic profiling of open chromatin, DNA-binding proteins and nucleosome position. *Nat Methods* **10**, 1213-1218 (2013).

10. Rizzetto, S. *et al.* B-cell receptor reconstruction from single-cell RNA-seq with VDJPuzzle. *Bioinformatics* **34**, 2846-2847 (2018).

11. Lun, A.T., Bach, K. & Marioni, J.C. Pooling across cells to normalize single-cell RNA sequencing data with many zero counts. *Genome Biol* **17**, 75 (2016).

12. Picelli, S. *et al.* Full-length RNA-seq from single cells using Smart-seq2. *Nature Protocols* **9**, 171-181 (2014).

13. Van Der Byl, W. *et al.* Single-Cell Transcriptome Analysis of T Cells. *Methods Mol Biol* **2048**, 155-205 (2019).

14. Bolger, A.M., Lohse, M. & Usadel, B. Trimmomatic: a flexible trimmer for Illumina sequence data. *Bioinformatics* **30**, 2114-2120 (2014).

15. Dobin, A. *et al.* STAR: ultrafast universal RNA-seq aligner. *Bioinformatics* **29**, 15-21 (2013).

16. Butler, A., Hoffman, P., Smibert, P., Papalexi, E. & Satija, R. Integrating single-cell transcriptomic data across different conditions, technologies, and species. *Nat Biotechnol* **36**, 411-420 (2018).

17. Aran, D. *et al.* Reference-based analysis of lung single-cell sequencing reveals a transitional profibrotic macrophage. *Nat Immunol* **20**, 163-172 (2019).

18. Miller, B.C. *et al.* Subsets of exhausted CD8(+) T cells differentially mediate tumor control and respond to checkpoint blockade. *Nat Immunol* **20**, 326-336 (2019).

19. Zheng, C. *et al.* Landscape of Infiltrating T Cells in Liver Cancer Revealed by Single-Cell Sequencing. *Cell* **169**, 1342-1356 e1316 (2017).

20. Jerby-Arnon, L. *et al.* A Cancer Cell Program Promotes T Cell Exclusion and Resistance to Checkpoint Blockade. *Cell* **175**, 984-997 e924 (2018).

21. Street, K. *et al.* Slingshot: cell lineage and pseudotime inference for single-cell transcriptomics. *BMC Genomics* **19**, 477 (2018).

22. Saelens, W., Cannoodt, R., Todorov, H. & Saeys, Y. A comparison of single-cell trajectory inference methods. *Nat Biotechnol* **37**, 547-554 (2019).

23. Dash, P. *et al.* Quantifiable predictive features define epitope-specific T cell receptor repertoires. *Nature* **547**, 89-93 (2017).

Figure S1, related to Figure 1

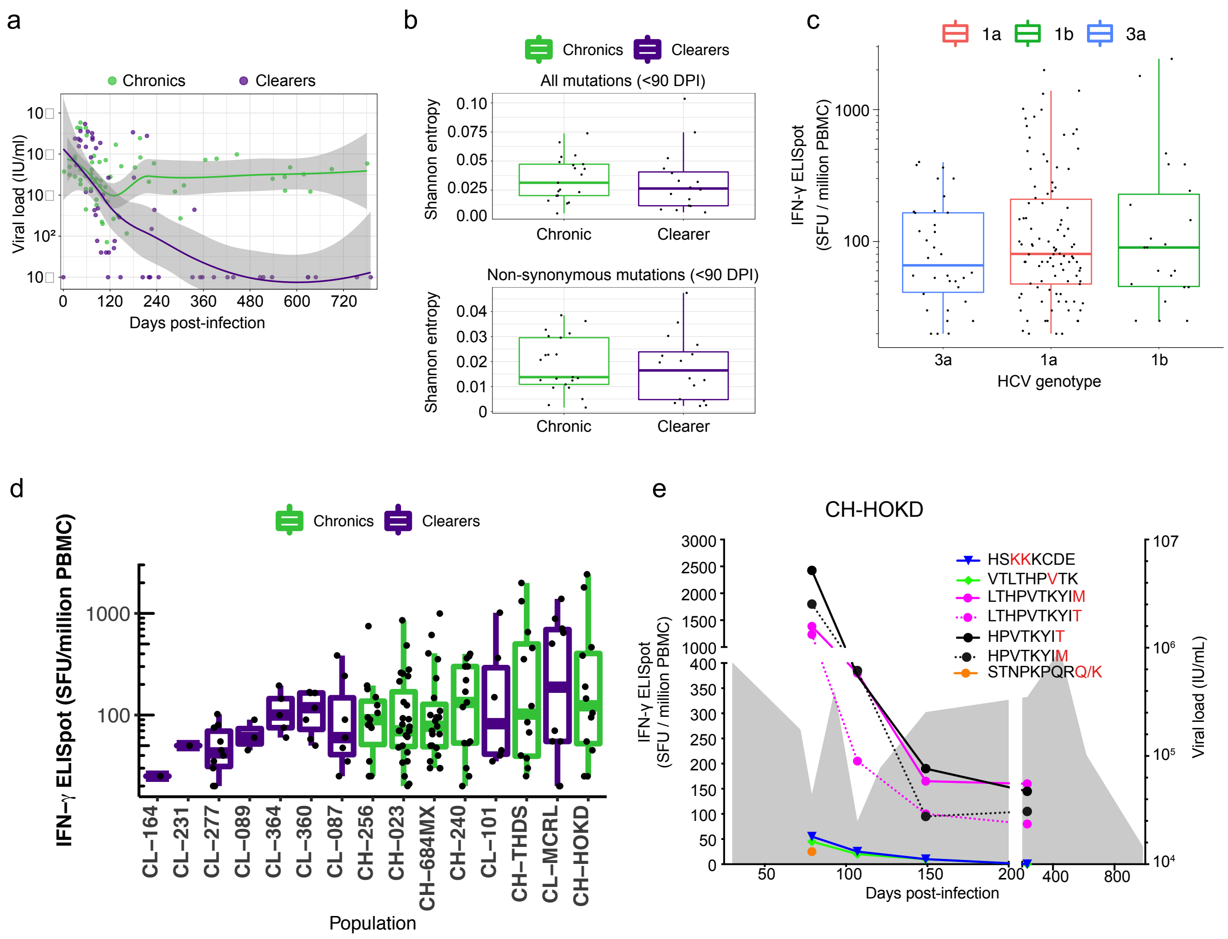

Supplementary Figure 1. Viral dynamics, genomic diversity, and magnitude of IFN- γ responses in acute phase of HCV infection.

(a) Kinetics of viral load. Each dot represents an individual sample time point across the 17 subjects included in this study. Lines represent the loess (locally estimated scatterplot smoothing) fit, and error bands represent 95% confidence intervals. **(b)** Comparison of viral genome diversity across disease outcomes ≤90 and >90 DPI. Diversity is measured via Shannon entropy from the frequencies of viral variants estimated from deep sequencing of HCV genomes at each viraemic time point. N=37 independent measures (sample time points) were used. Data are presented with box plots showing median and 75% quantile. Group comparison tests were performed utilizing two-sided Wilcoxon signed-rank test. **(c)** Comparison of IFN-γ ELISpot values by HCV genotype. Data are presented as median and 75%. Statistical test using two-sided Wilcoxon sum-rank test was not significant. **(d)** Distribution of IFN-γ ELISpot values by subjects. Data are presented as median and 75%. Data are presented as median and 75%. n=154 biologically independent measures were used in (c) and (d). **(e)** IFN- γ ELISpot values and vial load measured in subject CH-HOKD for 5 epitope-specific $CD8^+$ T cell responses. Of these, 2 were escape variants (dashed lines) of the original epitope found in the transmitted viral strain (transmitted founder virus). All the 5 epitopes revealed amino acid substitutions which eventually dominated the viral populations after 120 days post-infection.

Figure S2, related to Figure 2

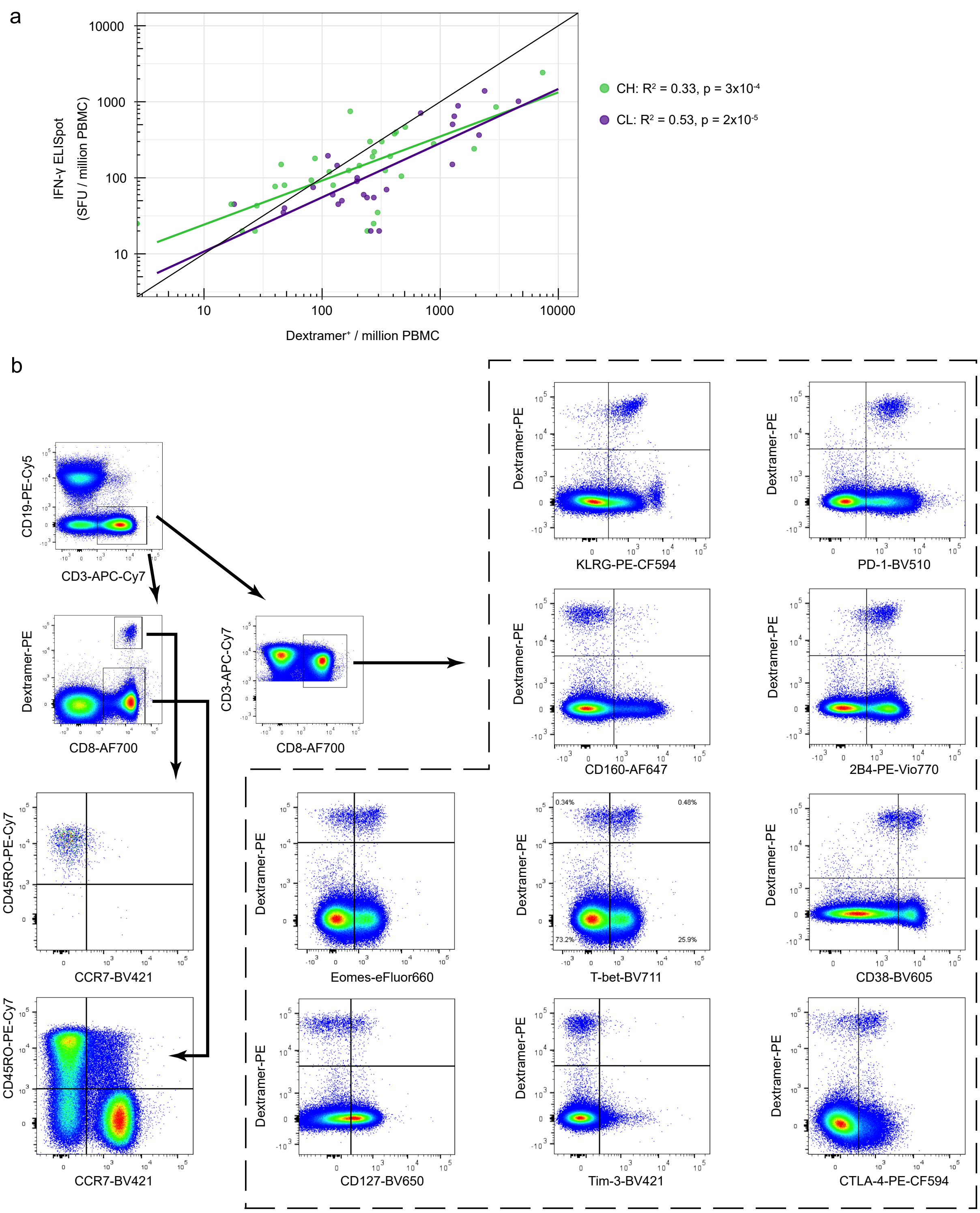

Supplementary Figure 2. Example of gating to identify HCV-specific $CD8^+$ T cell subsets from flow-cytometry data.

(a) Scatter plot showing the correlation between IFN-γ ELISpot values (number of SFU per million PBMC) and number of epitope-specific $CD8^+$ T cells (number of dextramer positive $CD8^+$ T cells per million PBMC). Linear regressions were obtained for both disease outcome. The black line represents the bisectrix, indicating a 1:1 correlation between both axes. CH: Chronic progressors, CL: Clearers. **(b)** Example of gating strategy adopted to identify phenotypic subsets of dextramer positive CD8+ T cell subsets. Data are obtained from two high dimensional flow cytometry panels (see Methods).

Figure S3, related to Figure 2

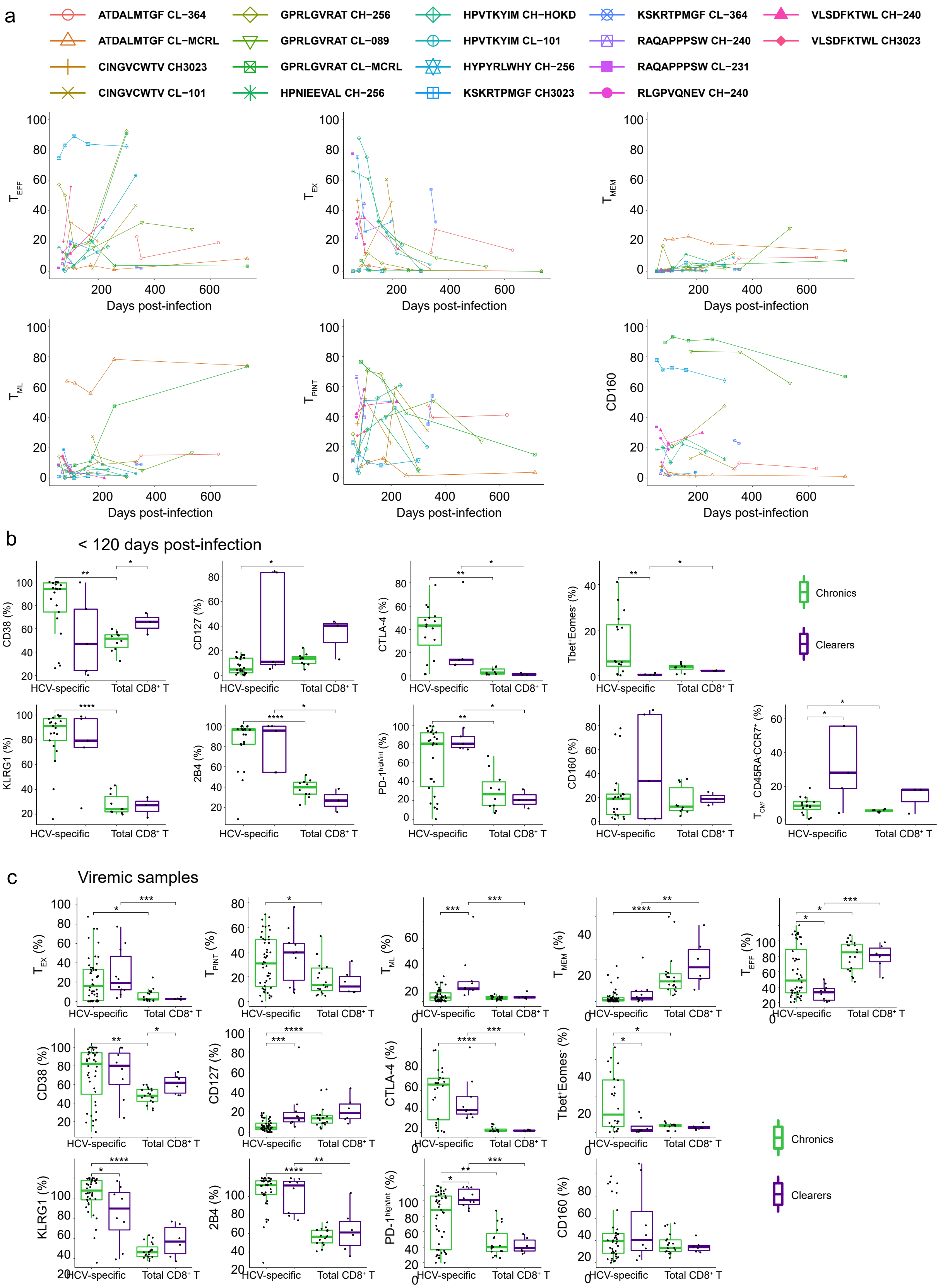

Supplementary Figure 3. Analysis of HCV-specific CD8+ T cell subsets over the course of the infection

(a) Proportion of subsets in HCV-specific CD8+ T cell samples over time, organised and coloured by epitope specificity and subject of origin. **(b-c)** Group comparison of the proportion of market specific T cell subsets between HCV-specific and total $CD8^+$ T cells in both disease outcomes. **(b)** early (≤120 DPI) and **(c)** only viraemic samples. Data are presented as box plot with median and 75% interval. Individual points represent populations from each subject's sample time points, and from each epitope specificity. n=79 biologically independent measures were used. Pairwise group comparisons were performed utilizing two-sided Wilcoxon signed-rank test. Statistical comparison performed with two-sided Wilcoxon Rank Sum Test (* $p < 0.05$, ** $p < 0.01$, *** $p < 0.001$, **** $p < 0.0001$).

Figure S4, related to Figure 2

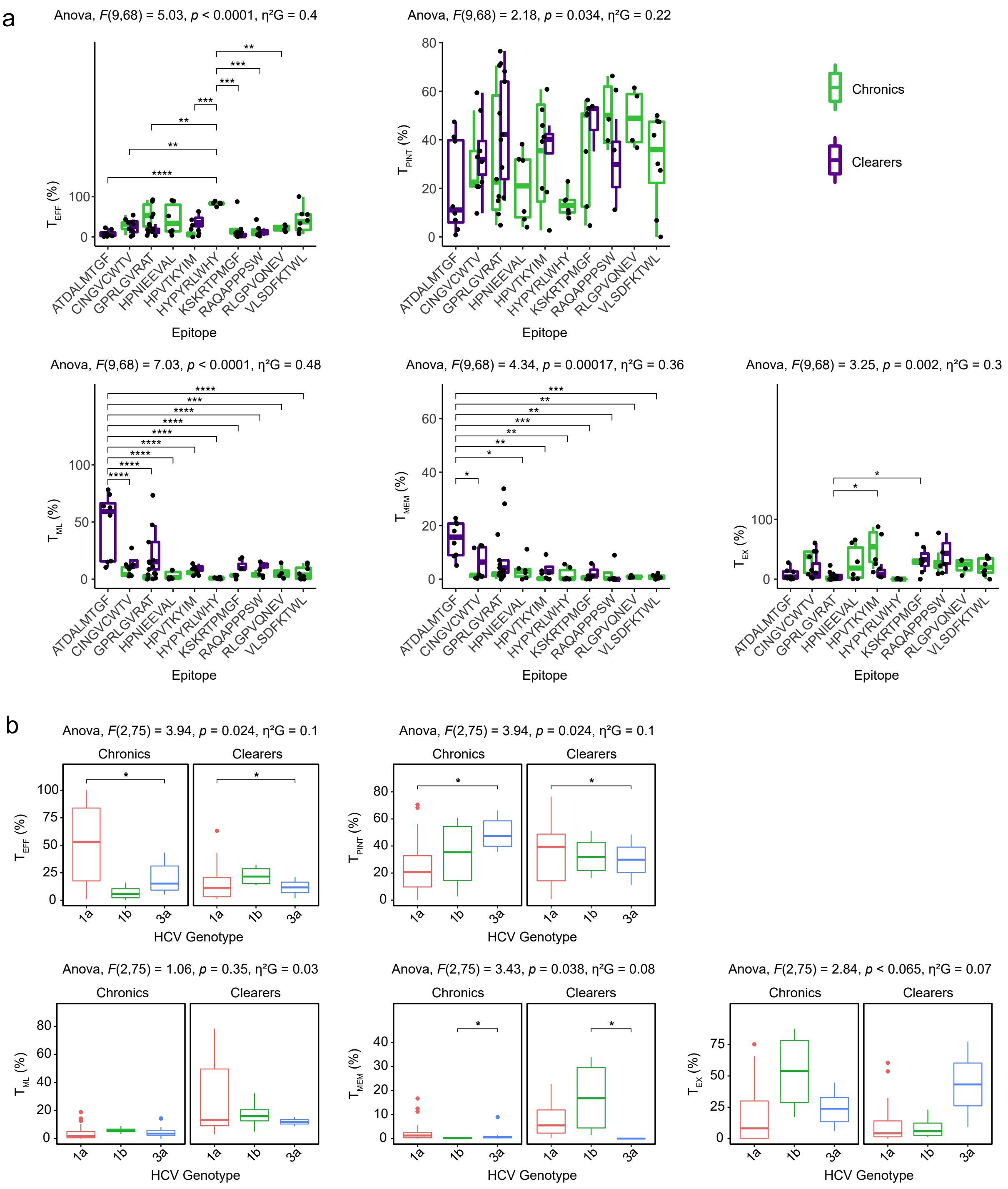

Supplementary Figure 4. Effect of HCV genotype and epitope specificity on the variation of phenotypic T cell subsets.

Analysis of variance (ANOVA), testing for the effect of epitope specificity **(a)** and HCV genotype **(b)**. Tukey's Honestly Significant Difference (Tukey's HSD) post-hoc test was performed for pairwise comparisons. F-test and generalized η^2 (to estimate effect size) are reported in each panel along with p-values (* $p < 0.05$, ** $p < 0.01$, *** $p < 0.001$, **** $p < 0.0001$). Data are presented with box plots showing median and 25%/75% quantiles. n=79 biologically independent measures were used.

Figure S5, related to Figure 2

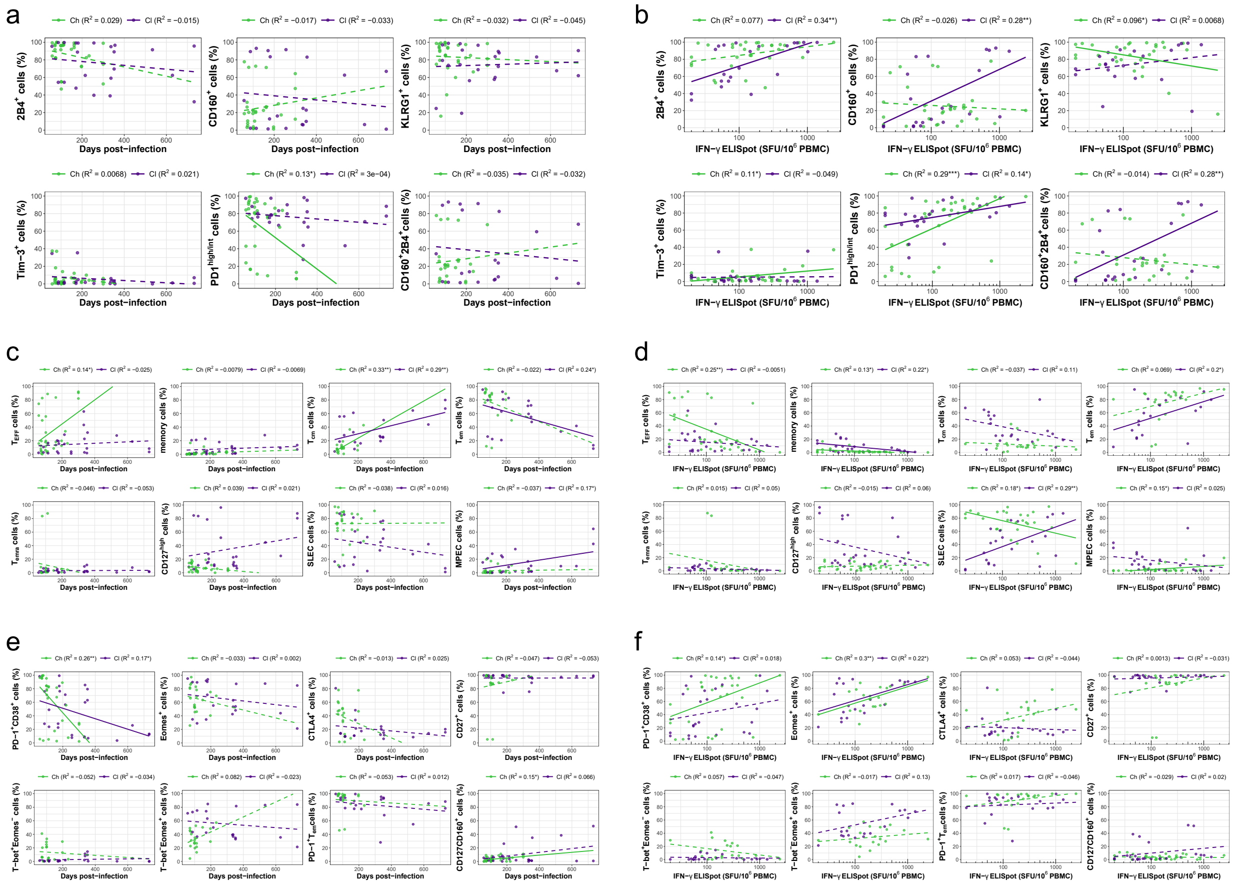

Supplementary Figure 5. Kinetics of T cell subsets over the course of infection and correlations with magnitude of IFN-*γ* response.

Scatter plots showing the relation between the proportion of dextramer positive $CD8^+$ T cell populations with positive expression of phenotypic markers (measured by flow cytometry) over the course of the infections (DPI values) with days post-infection (DPI) **(a, c, e)** or IFN-γ ELISpot values **(b, d, f)**. Shown are markers related to exhaustion **(a, b)**, memory **(c, d)** and differentiation **(e, f)**. Lines represent linear regression, adjusted coefficients and p-values are shown in the legend of each plot. Lines are dashed if insignificant (p-value > 0.05). (* $p < 0.05$, ** $p < 0.01$, *** $p < 0.001$, **** $p < 0.0001$).

Figure S6, related to Figure 3

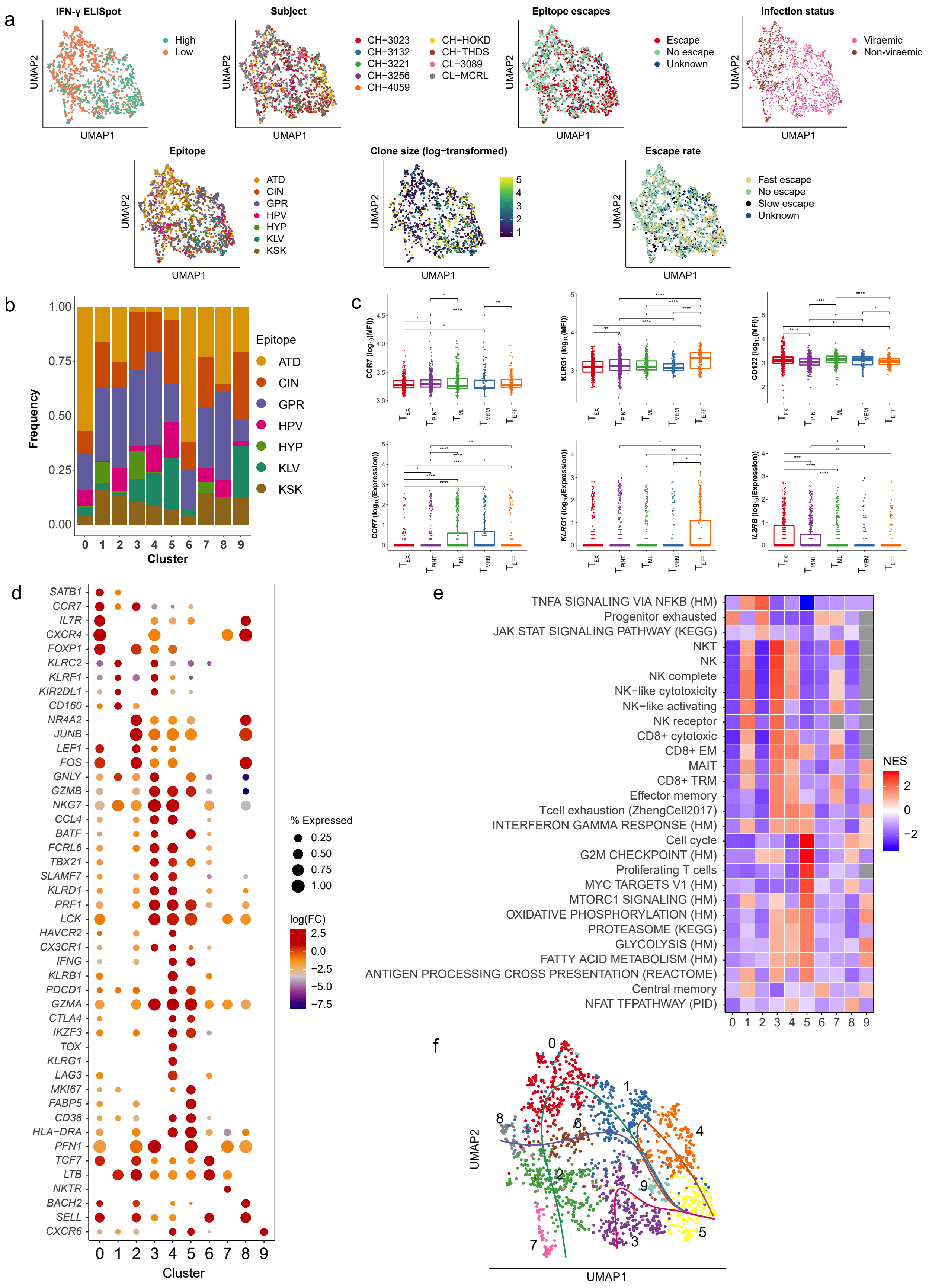

Supplementary Figure 6. Single-cell multi-omics uncover molecular and phenotypic heterogeneity of HCV-specific CD8$^+$ T cells.

(a) Dimensionality reduction (UMAP, as per Fig. 3b) of scRNA-seq data from HCV-specific CD8$^+$ T cells coloured by their IFN-γ ELISpot (high: ≥211, low: <211 SFU/Million PBMC), epitope specificity, subject of origin, epitope escape and specificity, clone size (log-transformed). **(b)** Distribution of epitope specificity across UMAP clusters (as per Fig. 3b). **(c)** Comparison of protein (log_{10}(scaled MFI + 1)) and corresponding gene (log_{10}(TPM + 1)) expression levels between T cell phenotypic subsets (n = 1603 cells from 26 biologically independent sample timepoints across 7 epitope specificities). Pairwise group comparisons were performed with two-sided Wilcoxon Rank Sum Tests (* $p < 0.05$, ** $p < 0.01$, *** $p < 0.001$, **** $p < 0.0001$). Shown are the median and 25%/75% quantiles. **(d)** Dot plot of selected genes identified from differential expression analysis (pairwise comparisons with a two-sided hurdle-model from MAST) between UMAP clusters. Dot size represents the proportion of cells with non-zero expression. **(e)** Heatmap of enriched pathways identified from GSEA using differentially expressed genes (as per (d)) between UMAP clusters. All pathways shown have adjusted p-values < 0.05 in at least one cluster. NES: normalized enrichment score. **(f)** Trajectories obtained using Slingshot projected onto the UMAP (as per Fig. 3b), root was chosen in cluster 5.

Figure S7, related to Figure 3

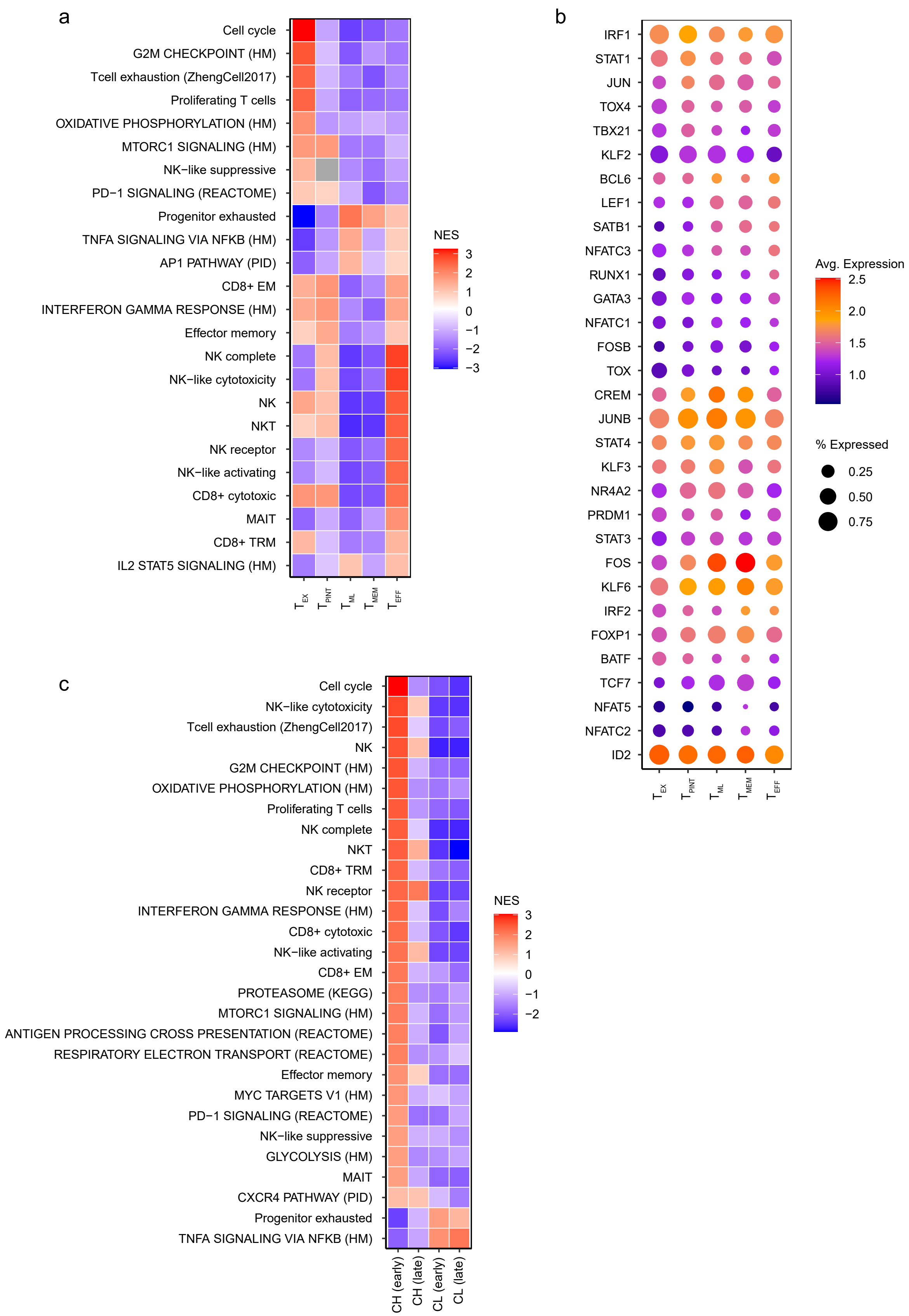

Supplementary Figure 7. Gene expression profiles in cells from chronic infections

(a) Heatmap of enriched pathways identified from GSEA using differentially expressed genes between T cell phenotypes. All pathways shown have adjusted p-values < 0.05 in at least one phenotype. NES: normalized enrichment score. **(b)** Dot plot of selected transcription factors grouped by disease outcome. Dot size represents the proportion of cells with non-zero expression from each phenotype. The colour represents average mean expression. CH: Chronic progressors, CL: Clearers. **(c)** Heatmap of enriched pathways identified from GSEA using differentially expressed genes between early and late phases of chronic progressors (CH) and clearers (CL). All pathways shown have adjusted p-values < 0.05 in at least one disease outcome/stage group. NES: normalized enrichment score.

Figure S8, related to Figure 3

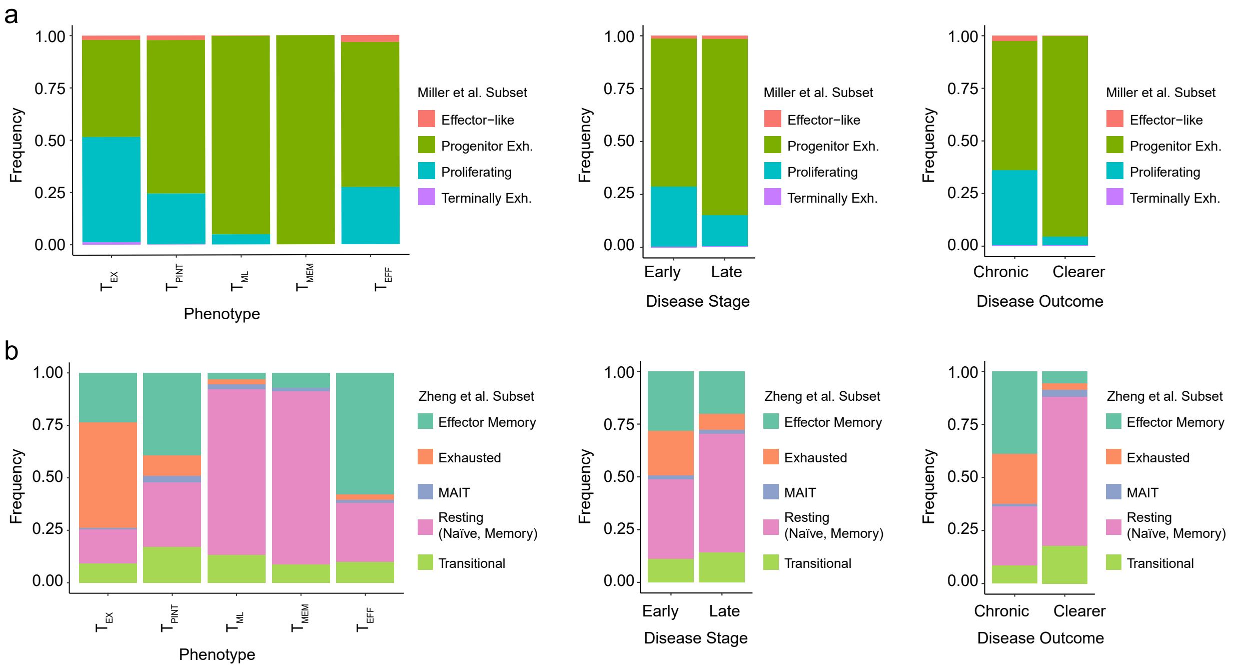

Supplementary Figure 8. Reference-based analysis of scRNA-seq profiles of T cell phenotypes.

(a) Distribution of Miller et al.[28] subsets (in colours) across cells organised by T cell phenotype, disease stage (Early: ≤120 DPI, Late: >120 DPI) and disease outcome. **(b)** Distribution of Zheng et al.[29] subsets across cells organised by T cell phenotype, disease stage (Early: ≤120 DPI, Late: >120 DPI) and disease outcome.

Figure S9, related to Figure 4

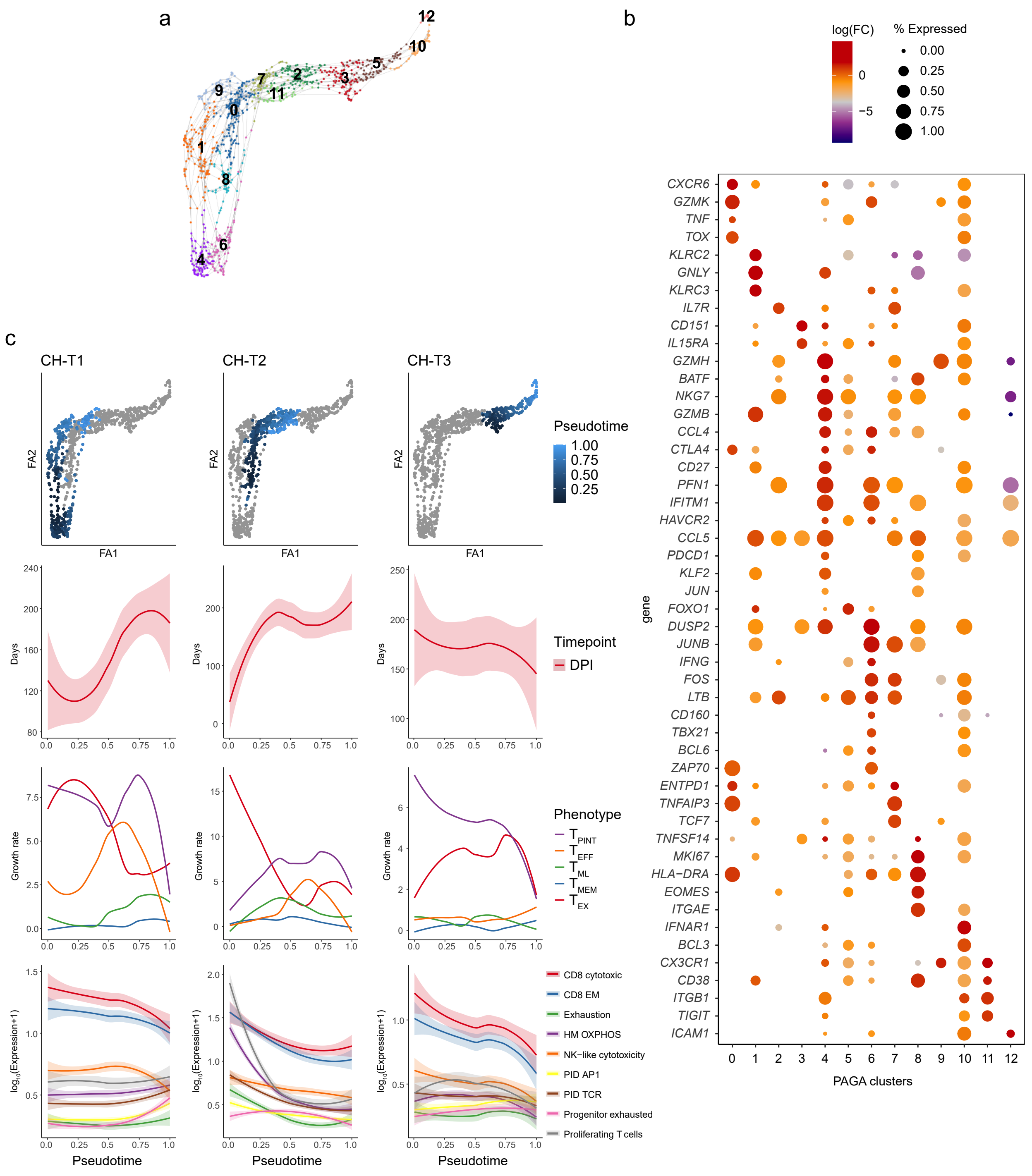

Supplementary Figure 9. Distinct evolutionary trajectories identified from single cell transcriptomics explain functional heterogeneity in clearers.

(a) PAGA graphs and clusters of single cells (n=903) identifying 13 clusters and their probability of connection (thickness of lines). **(b)** Dot plot of selected genes identified from differential expression analysis between PAGA clusters of cells from chronic infections. Only significant genes are shown ($p < 0.05$, $|\log_2(FC)| \geq 0.3$). The ball size represents the proportion of cells with non-zero expression. The colour represents log-fold-change relative to cells from other clusters. **(c)** Diffusion-pseudotime plots with colour gradients identifying three evolutionary trajectories (CH-T1, CH-T2 and CH-T3), as in Fig. 4e. Loess curves below show the DPI and growth rates estimated from the calculated size of T cell phenotypes over pseudotime for each trajectory. Bottom three loess curves show average gene expression of genes in selected gene signatures.

Figure S10, related to Figure 4

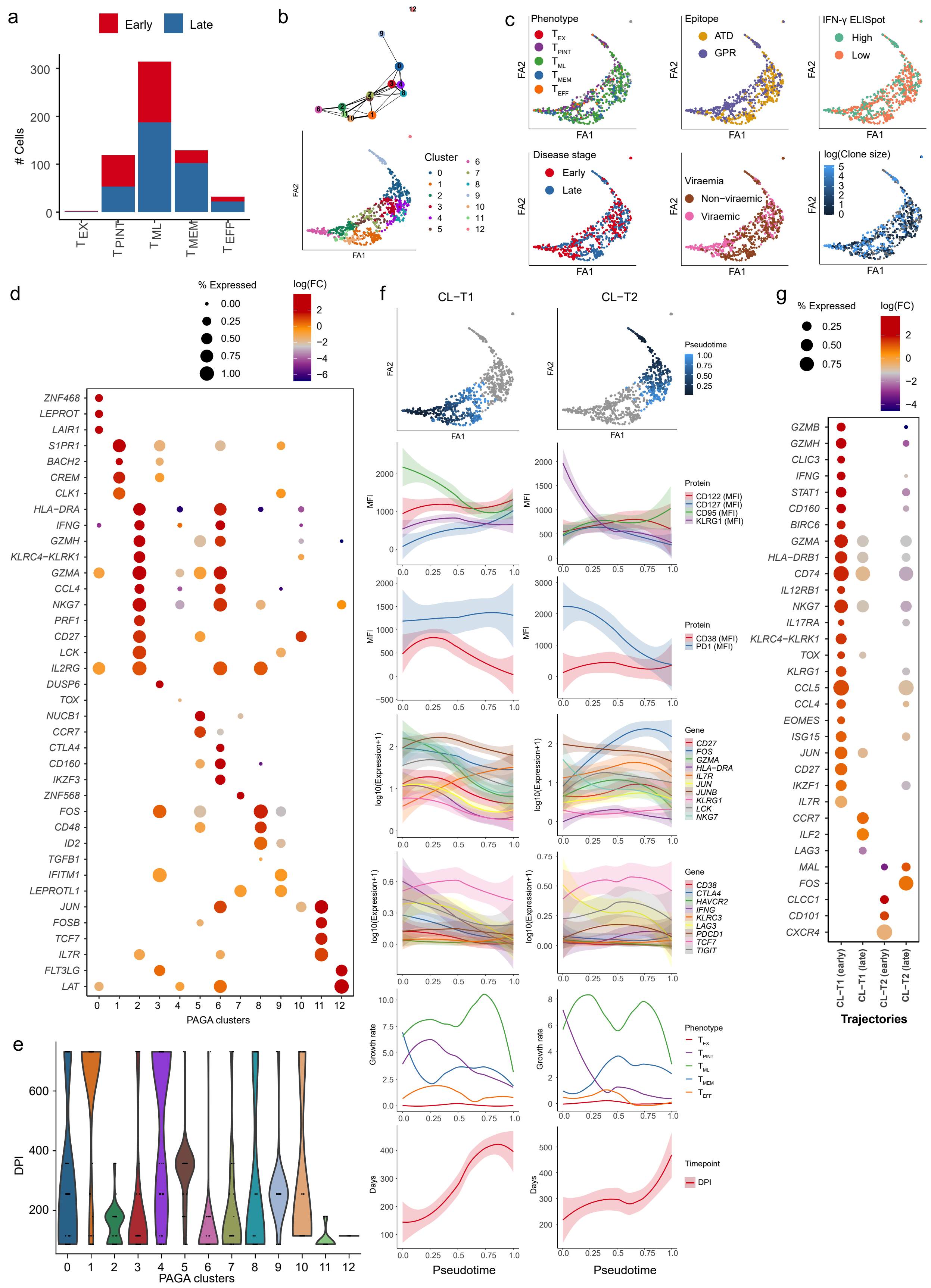

Supplementary Figure 10. Trajectory analysis in clearers revealed maintenance of T_{PINT} and increase of T_M and T_{ML}.

(a) Distribution of single cells from clearers by phase of infection (Early: ≤120 DPI, late: >120 DPI) in each T cell phenotype. **(b)** PAGA graphs of single cells from clearers (n=700 cells). The graph revealed both static and dynamic relationships by identifying 13 clusters of cells as nodes and their connectivity (quantified by line weight as edges between clusters). **(c)** PAGA graphs (visualised using ForceAtlas2 layout algorithm (FA1, FA2)) coloured by sample viremia, epitope specificity, disease outcome, disease stage, T cell phenotype, IFN-γ ELISpot (high: ≥211, low: <211 SFU/Million PBMC) and clone size (log-transformed). **(d)** Dot plot of selected differentially expressed genes between the PAGA clusters. Only significant genes are shown ($p < 0.05$, $|\log_2(FC)| \geq 0.5$). The ball size represents the proportion of cells with non-zero expression. The colour represents log-fold-change relative to cells from other clusters. **(e)** Violin plot of DPI distribution in each PAGA cluster. **(f)** Diffusion-pseudotime plots with colour gradients identifying two evolutionary trajectories (CL-T1 and CL-T2). Loess curves below show the expression of proteins and genes over the pseudotime in each trajectory, as well as the DPI and growth rates estimated from the calculated size of T cell phenotypes over pseudotime for each trajectory. **(g)** Dot plot of selected differentially expressed genes between the early and late phases of each trajectory. Only significant genes are shown ($p < 0.05$, $|\log_2(FC)| \geq 0.5$). The ball size represents the proportion of cells with non-zero expression. The colour represents log-fold-change relative to cells from the same trajectory at early or late phases. Early ≤120 DPI, late > 120 DPI.

Supplementary Table 1. Clinical and laboratory characteristics of the subjects (N=17) included in this study.

Subjects	Age at infection /Sex	Symptomatic	HLA type			HCV genotype	No. of days post infection [a]	HCV Ab	HCV RNA	Viral Load (IU/ml)	Number of epitopes detected
			**A**	**B**	**C**						
CH-240	21/M	Yes	0201	1501	0304	3a	-61	-	-	0	
			0201	5701	0602		**44**	-	+	54,887	3
							57	+	+	85,473	
							71	+	+	64,063	3
							85	+	+	6,051	3
							99	+	+	497	3
							113	+	+	4,862	3
							**140**	+	+	1,034	3
							**220**	+	+	44,449	3
							310	+	+	29,552	3
							**538**	+	+	62,174	3
CH-023	22/M	Yes	0201	4402	0501	1a	-165	-	-	0	
			0201	5701	0602		**36**	-	+	19,234,348	8
							**44**	-	+	17,907,338	8
							**60**	+	+	8,121,396	8
							**74**	+	+	397,185	8
							85	+	+	3,218	
							101	+	+	398	8
							**135**	+	+	2,843,176	8
							**197**	+	+	5,896,155	8
							365	+	+	516,945	8
						3a	635	+	+	14,738	8
							781	+	+	344,388	
CH-HOKD	26/F	Yes	0101	0801	0602	1b	-135	-	-	0	
			3001	1302	0701		**30**	-	+	733,849	
							65	NA	NA		
							**72**	+	+	175,219	
							**79**	+	+	44,452	5
							**93**	+	+	407,392	
							**107**	+	+	24,969	5
							**121**	+	+	77,723	
							**149**	+	+	254,245	5
							170	+	NA		

							**233**	+	+	350,658	5
						1a	446	+	+	956,488	
							618	+	+	102,439	
							985	+	+	14,587	
CH-256	30/M	No	0301	0702	0401	1a		-	-	0	
			2402	3501	0702		**44**	-	+	34,149,824	
							**58**	+	+	19,188,762	6
							**79**	+	+	812,622	6
							**96**	+	+	50,774	
							112	+	+	50	6
							128	+	+	693	
							162	+	+	135	6
							**286**	+	+	14,853	6
							300	+	+	732	
							569	+	+	16,421	
							944	+	+	85,835	
CH-THDS	25/M	No	0201	1402	0102	1a	-152	-	-	0	
			3201	2705	0802		**16**	-	+	235,662	
							30	-	+	549,251	
							44	-	+	176,550	
							58	+	+	2,005	
							72	+	+	108,737	3
							85	+	+	62,043	
							**109**	+	+	503,981	
							**198**	+	+	681,389	
							395	+	+	223,849	
							530	+	+	183,426	
CH-684MX	27/M	Yes	0201	2702	0202	1a	-166	-	-	0	
			0301	4001	0304		**2**	-	+	140,200	
							16	-	+	98,867	
							30	-	+	78,764	
							44	-	+	33,763	
							**58**	+	+	19,932	
							71	+	+	20,374	5
							95	+	+	28,181	
							**184**	+	+	221,964	
							380	+	+	75,688	
							515	+	+	143,746	
CL-360	28/M	Yes	3201	1402	0501	3a	-110	-	-	0	
			6802	4402	0802		**30**	-	+	5,648,631	

							**44**	-	+	4,617,483	3
							58	+	+	14,170	
							**71**	+	+	15,938	3
							83	+	+	1,060	
							97	+	+	<15	3
							132	+	+	57	
							223	+	-	0	
							422	+	-	0	3
CL-277	24/M	No	0201	4402	0501	3a	-578	-	-	0	
			1101	4402	0501		**39**	-	+	5,482,503	
							63	+	+	12,442,419	
							**74**	+	+	10,374,554	
							95	+	+	3,473,088	
							**102**	+	+	10,506	
							**116**	+	+	70,120	6
							144	+	+	833	
							245	+	-	0	
							437	+	-	0	
CL-MCRL	25/F	No	0101	0702	0702	1a	-81	-	-	0	
			2902	4403	1601		80	+	+	1,846	
							87	+	+	58	2
							115	+	-	0	2
							171	+	-	0	
							256	+	-	0	2
							487	+	-	0	
							648	+	-	0	2
							732	+	-	0	
CL-087	32/F	NA	2402	1402	0403	1b	-46	-	-	0	
			3004	1506	0802		**31**	+	+	13,118,082	
							42	+	+	29,257,428	
							61	+	+	6,455,009	
							**70**	+	+	1,940,469	
							116	+	+	25	3
							133	+	-	0	
CL-364	29/M	Yes	0101	0702	0602	1a	-337	-	-	0	
			0301	5701	0702		337	+	+	1,932	2
							352	+	-	0	2
							629	+	-	0	2

CL-231	22/M	NA	0101	0702	0602	3a	**57**	+	+	2,242,163	1
			0101	5701	0702		199	+	-	0	1
CL-089	26/M	No	0101	0702	0501	1b	-180	-	-	0	
			3001	4402	0702		**180**	+	+	70,737	1
							356	+	-	0	1
							534	+	-	0	1
							752	+	-	0	1
Cl-101	36/M	NA	0101	0801	0501	1a/3a	-536	-	-	0	
			0201	4402	0701		178	+	+	11441811	2
							215	+	+	7646402	2
							333	+	+	0	2
							385	+	+	0	2
CH-221	21/M	NA	1101	0702	0102	3a	46.5	+	+	315892	1
			3001	4601	0702		523.5	+	+	200219	
							787.5	+	+	294540	
CH-132	20/F	NA	0101	0801	0303	3a	-262.5	-	-	0	1
			3101	5501	0701		262.5	+	+	26678	
							472.5	+	+	65365	
							691.5	+	+	118153	
							842.5	+	+	164179	
							1564.5	+	+	73491	
CH-059	30/M	NA	0101	0702	0701	1a	-80.5	-	-	0	1
			0201	0801	0702		13.5	-	+	3676682	
							27.5	-	+	17409803	
							41.5	NA	+	26187596	
							60.5	+	+	7308585	
							76.5	+	+	488992	
							88.5	+	+	5906	
						2	102.5	+	+	588467	
							195.5	+	+	1096633	
							423.5	+	+	321276	

[a] The number of days post infection was estimated from the time to seroconversion.
Bold denotes time points with viral samples sequenced for detection of autologous virus using Next Generation Sequencing.

Supplementary Table 2. Viral sequencing and epitopes detected and tested in ELISPOT assays.

Subject	**Outcome of infection**	**Geno-type**	**Viral genome sequence (Sanger)**	**Deep Sequencing (NGS)**	**Number of epitopes tested in ELISPOT***
CH-240	Chronic	3a	+	+	70
CH-023	Chronic	1a	+	+	45
CH-HOKD	Chronic	1b	+	+	82
CH-256	Chronic	1a	+	+	98
CH-THDS	Chronic	1a	+	+	99
CH-684MX	Chronic	1a	+	+	100
CL-360	Clearer	3a	+	+	93
CL-277	Clearer	3a	+	+	69
CL-MCRL	Clearer	1a	+	-	3
CL-087	Clearer	1b	+	+	71
CL-364	Clearer	1a	+	+	8
CL-231	Clearer	3a	+	+	41
CL-089	Clearer	1b	+	-	5
CL-101	Clearer	1a,3a	+	-	9
Ch-221	Clearer	3a	-	-	1*
Ch-059	Chronic	1a	+	+	1*
Ch-132	Chronic	3a	-	-	0

*Tested with negative results

Supplementary Table 3. HLA-I restricted T-cell epitopes identified with positive IFN-γ ELISpot responses for each subject.

Subject	HLA-restriction	Epitope [a]	Position (aa)[a]	Protein
**CH-3240**	HLA-B*57:01	RAQAP(L)PPSW	1602–1610	NS3
	HLA-A*02:01	RLGPVQNEV(I)	1633–1641	NS3
	HLA-A*02:01	VLSDFKT(A)WL	1992-2000	NS4B
**CH-3023**	HLA-B*44:02	AEVIAPAVQT	1743–1752	NS4B
	HLA-B*57:01	FAWYLKGKW	774–782	E2
	HLA-B*57:01	K(N)SKRTPMGF	2629–2637	NS5B
	HLA-B*57:01	RAEAQ(H)LHAW	852–860	NS2
	HLA-B*44:02	AELIEANLLW	2228–2237	NS5A
	HLA A*02:01	WLGNIIMFA	2827–2835	NS5B
	HLA-A*02:01	CINGVCWTV	1073-1081	NS3
	HLA-A*02:01	VLSDFKTWL	1992-2000	NS4B
**CH-HOKD**	HLA-B*0801	HPVTKYIM(T)	1639-1646	NS3
	HLA-A*3001	LTHPVTKYIM	1637-1646	NS3
	HLA-A*0101	VTLTHPV(I)TKY	1635-1644	NS3
	HLA-A*0101	STNPKPQRK(Q)	2-10	CORE
	HLA-B*0801	HSKK(RR)KCDEL	1395- 1403	NS3
**CH-3256**	HLA-B*07:02	GPRL(KM)GVRAT	41-49	CORE
	HLA-A*03:01	H(D)YPYRLWHY	610-618	E2
	HLA-B*35:01	HPN(S)IEEVAL	1359-1367	NS3
	HLA-B*35:01	HAVGIFRAA	1175-1183	NS3
	HLA-B*35:01	YGKAIPLEVI	1376-1385	NS3
	HLA-A*03:01	ALGVNAVAYY	1409-1418	NS3
**CH-THDS**	HLA-B*27:05	A(V)RMVMMTHF	2842-2850	NS5B
	HLA-A*02:01	KLVAMGI(L)NAV	1406-1415	NS3
	HLA-A*02:01	TLSPYYKRY(H)I	830-839	NS2
**CH-684MX**	HLA-A*02:01	SILGIGTA(V)L	1325-1333	NS3
	HLA-A*02:01	AWETARH(Y)TPV	2816-2825	NS5B
	HLA-A*02:01	KLVAMGINAV	1406-1415	NS3
	HLA-B*27:05	A(V)RMVMMTHF	2841-2849	NS5B
	HLA-A*02:01	TSILGIGTA(V)	1324-1332	NS3
**CL-360**	HLA-A*32:01	YLTAYQATV	1591–1599	NS3
	HLA-A*68:02	SVIDCNVAV	1456–1464	NS3
	HLA-A*68:02	ATDALMTGF	1436-1444	NS3
**CL-277**	HLA-A*02:01	NLPGCSFSI	168-176	CORE
	HLA-A*02:01	RLWHYPCTV	620-628	E2
	HLA-A*11:01	HSNIEEVAL	1365-1373	NS3
	HLA-A*02:01	ILAGYGAGV	1857-1865	NS4B
	HLA-A*02:01	AWETARHTPV	2826-2835	NS5B
	HLA-A*02:01	WLGNIIMYA	2838-2846	NS5B
**CL-MCRL**	HLA-B*07:02	GPRLGVRAT	41-49	CORE
	HLA-A*0101	ATDALMTGF	1436-1444	NS3
**CL-087**	HLA-A*24:02	VHYPYRLWHY	610-619	E2
	HLA-A*24:02	GAPITYSTY	1289-1297	NS3
	HLA-A*24:02	SFSIFLLAL	173-181	CORE
**CL-364**	HLA-B*57:01	KSKRTPMGF	2629–2637	NS5B
	HLA-A*0101	ATDALMTGF	1436-1444	NS3
**CL-231**	HLA-B*57:01	RAQAPPPSW	1602–1610	NS3
**CL-3089**	HLA-B*07:02	GPRLGVRAT	41-49	CORE
**CL-101**	HLA-A*02:01	CINGVCWTV	1073-1081	NS3
	HLA-B*0801	HPVTKYIM	1639-1646	NS3
**CH-3221** [b]	HLA-B*07:02	GPRLGVRAT	41-49	CORE
**CH-4059** [b]	HLA-A*02:01	CINGVCWTV	1073-1081	NS3
**CH-3132** [b]	HLA-A*0101	ATDALMTGF	1436-1444	NS3

[a] Amino acids in red correspond to mutations that become dominant (>75% of the viral population).
[b] No viral sequencing available for these epitopes. IFN-γ responses not tested.

Supplementary Table 4. Estimates of the rate of escape for viral epitopes

Subject ID	Epitope (escape variant)*	Epitope (WT)	IFN-γ ELISPOT[&]	Rate of escape (Std Error)	P-value
CH-HOKD	HPVTKYIT	HPVTKYIM	2425	0.387 (0.074)	0.002
	LTHPVTKYIT	LTHPVTKYIM	1385	0.387 (0.074)	0.002
	HSRRKCDEL	HSKKKCDEL	55	0.245 (0.027)	0.0001
CH-THDS	KLVAMGLNAV	KLVAMGINAV	1992.5	0.330 (0.037)	0.0009
	TLSPYYKRHI	TLSPYYKRYI	67.5	0.085 (0.014)	0.0036
	VRMVMMTHF	ARMVMMTHF	655	0.160 (0.074)	0.0700
THGS0684MX	TSILGIGTV	TSILGIGTA	230	0.350 (0.372)	0.4000
	SILGIGTVL	SILGIGTAL	997.5	0.350 (0.213)	0.4000
	AWETARYTPV	AWETARHTPV	50	0.080 (0.008)	0.0087
CH-3256	DYPYRLWHY	HYPYRLWHY	750	0.560 (0.039)	0.0007
	GPKMGVRAT	GPRLGVRAT	195	0.047 (0.003)	0.0050
	HPSIEEVAL	HPNIEEVAL	150	0.049 (0.002)	0.0000
CH-3023	RAEAHLHAW	RAEAQLHAW	261	0.080 (0.005)	0.0001
	NSKRTPMGF	KSKRTPMGF	854	0.166 (0.035)	0.0090
CH-3240	RAQALPPSW	RAQAPPPSW	220	0.102 (0.016)	0.0080
	RLGPVQNEI	RLGPVQNEV	380	0.003 (0.002)	0.0007

*Dominant escape variant (i.e., frequency of occurrence >70%)
& Maximum value of SFU per million PBMC against wild type epitope measured within the first 120DPI.

Supplementary Table 5. HLA class I dextramers used for immunophenotyping of HCV-specific CD8+ T cells.

HCV protein	Position (aa)	Epitope sequence	WT/escape	T cells identified	HLA-I	Subjects
NS3	1602–1610	RAQAPPPSW	WT	Y	B*57:01	CH-3240; CL-231
NS3	1602–1610	RAQALPPSW	ESCAPE	N	B*57:01	CH-3023
NS3	1633–1641	RLGPVQNEV	WT	Y	A*02:01	CH-3240
NS3	1633–1641	RLGPVQNEI	ESCAPE	N	A*02:01	CH-3240
NS4B	1992-2000	VLSDFKTWL	WT	Y	A*02:01	CH-3023; CH-3240
NS4B	1992-2000	VLSDFKAWL	ESCAPE	N	A*02:01	CH-3023; CH-3240
NS5B	2629–2637	KSKRTPMGF	WT	Y	B*57:01	CH-3023; CL-364
NS5B	2629–2637	NSKRTPMGF	ESCAPE	N	B*57:01	CH-3023
NS2	852–860	RAEAQLHAW	WT	N	B*57:01	CH-3240
NS3	1073-1081	CINGVCWTV	WT	Y	A*02:01	CH-3023; CH-4059; CL-101
NS3	1639-1646	HPVTKYIM	WT	Y	B*0801	CL-101; CH-HOKD
NS3	1395- 1403	HSKKKCDEL	WT	N	B*0801	CH-HOKD
E2	610-618	HYPYRLWHY	WT	Y	A*03:01	CH-3256
CORE	41-49	GPRLGVRAT	WT	Y	B*07:02	CH-HOKD; CH-3256; CH-3221; CL-3089

NS3	1359-1367	HPNIEEVAL	WT	Y	B*35:01	CH-3256
NS3	1175-1183	HAVGIFRAA	WT	N	B*35:01	CH-3256
NS3	1406-1415	KLVAMGINAV	WT	N	A*02:01	CH-684MX
NS3	1436-1444	ATDALMTGF	WT	Y	A*0101	CL-364; CL-3132; CL-MCRL
NS3	1436-1444	ATDALMTGY	ESCAPE	N	A*0101	CL-364
NS5B	2827–2835	WLGNIIMFA	WT	N	A*02:01	CH-3023

Supplementary Table 6. TCR detection (includes VDJPuzzle TCR reconstruction from scRNA-seq data, and sanger sequencing)

Subject	Epitope	Number of cells with TCRαβ (%)	Unique CDR3αβ	Expanded CDR3αβ clones*	Number of cells with CDR3α	Number of unique CDR3α	Number of expanded CDR3α clones	Number of cells with CDR3β	Number of unique CDR3β	Number of expanded CDR3β clones
CL-MCRL	GPR	174 (71%)	2	2	181	2	2	228	3	2
CL-MCRL	ATD	262 (80%)	129	46	275	111	45	304	129	59
CL-3089	GPR	98 (75%)	44	16	100	41	17	125	42	23
CH-THDS	KLV	116 (97%)	22	10	116	21	10	118	21	10
CH-HOKD	HPV	99 (92%)	63	16	100	52	19	107	65	19
CH-4059	CIN	83 (70%)	59	11	88	59	12	105	75	15
CH-3256	HYP	37 (55%)	4	1	38	4	1	66	2	1
CH-3256	GPR	14 (78%)	5	3	14	5	3	18	4	2
CH-3240	RLG	143 (99%)	22	7	144	19	7	143	10	7
CH-3240	RAQ	123 (98%)	64	13	126	55	13	123	13	8
CH-3221	GPR	63 (70%)	10	4	65	9	4	87	12	5
CH-3132	ATD	26 (62%)	20	4	30	23	4	36	28	4
CH-3023	CIN	130 (65%)	117	6	137	118	11	186	162	11
CH-3023	KSK	195 (71%)	100	38	198	96	40	267	86	42

* Number of CDR3αβ clones that are observed more than once

Supplementary Table 7. Fluorophore-conjugated antibodies used in this study.

Target	Fluorophore	Clone	Vendor	Catalogue number	Dilution
CD4	FITC	RPA-T4	BD	555346	1:20
CD19	PE-Cy5	HIB19	BD	555414	1:20
CD3	APC-Cy7	SK7	BD	557832	1:10
CD8	AF700	RPA-T8	BD	561026	1:10
PD-1	BV510	EH12.1	BD	563076	1:20
TIM-3	BV421	F38-2E2	BioLegend	345008	1:20
TIM-3	PerCP-Cy5.5	F38-2E2	BioLegend	345016	1:20
CD38	BV605	HB7	BD	562665	1:20
CD127	BV650	HIL-7R-M21	BD	563225	1:20
2B4	PE-Vio770	REA112	Miltenyi Biotec	130-099-074	1:20
CD160	AF647	BY55	BioLegend	341204	1:20
Dextramer	PE	Various	Immudex	Various	1:20
KLRG1	Biotin	2F1	Biolegend	138406	1:100
Streptavidin	PE-CF594	None	BD	562284	1:100
CCR7	BV421	150503	BD	562555	1:20
CD45RO	PE-Cy-7	UCHL1	BD	337168	1:20
CD27	BUV395	L128	BD	563816	1:20
CTLA-4	PE-CF594	BNI3	BD	562742	1:20
T-bet	BV711	O4-46	BD	563320	1:20
EOMES	eFluor660	WD1928	eBioscience	50-4877-42	1:20
CD3	BV480	UCHT1	BD	566105	1:50
CD122	BV650	MiK-β3	BD	743117	1:40
CD95	BV786	DX2	BD	740991	1:80
CD38	APC	HB7	BD	345807	1:20
CD8	APC-R700	RPA-T8	BD	565165	1:10
PD-1	PE-CF594	EH12.1	BD	565024	1:20
CD127	PE-Cy7	HIL-7R-M21	BD	560822	1:10
CD45RA	FITC	HI100	BD	555488	1:5
KLRG1	PerCP-Cy5.5	SA231A2	BioLegend	367708	1:20
CXCR3	APC	1C6/CXCR3	BD	550967	1:20
