# Supplementary material for: Identification of human progenitors of exhausted CD8+ T cells associated with elevated IFN-γ response in early phase of viral infection
Source: Nat Commun. 2022 Dec 7;13:7543. doi: 10.1038/s41467-022-35281-7 (PMC9729230; doi:10.1038/s41467-022-35281-7)
Supplement: Supplementary file 3 — Description of Additional Supplementary Files [file 41467_2022_35281_MOESM3_ESM.pdf]

Description of Additional Supplementary Files

File Name: Supplementary data 1

Description: ELISpot data for each epitope specific CD8+ T cells.

File Name: Supplementary data 2

Description: Statistical analysis of the effect of epitope specificity and viral genotype on T-cell phenotypes.

File Name: Supplementary data 3

Description: Metadata file of single cells, including full length TCR sequences.

File Name: Supplementary data 4

Description: Differentially expressed genes between groups of cells or trajectories

File Name: Supplementary data 5

Description: Customised gene signatures utilised for the GSEA.

File Name: Supplementary data 6

Description: GSEA results between groups of cells or trajectories.

File Name: Supplementary data 7

Description: Variable opening chromatin regions (ATAC-seq results) between the epitope specific T-cell responses.
