# Supplementary material for: Identification of human progenitors of exhausted CD8+ T cells associated with elevated IFN-γ response in early phase of viral infection
Source: Nat Commun. 2022 Dec 7;13:7543. doi: 10.1038/s41467-022-35281-7 (PMC9729230; doi:10.1038/s41467-022-35281-7)
Supplement: Supplementary file 11 — Reporting Summary [file 41467_2022_35281_MOESM11_ESM.pdf]

nature portfolio

Corresponding author(s): Fabio Luciani

Last updated by author(s): Aug 22, 2022

Reporting Summary

Nature Portfolio wishes to improve the reproducibility of the work that we publish. This form provides structure for consistency and transparency in reporting. For further information on Nature Portfolio policies, see our Editorial Policies and the Editorial Policy Checklist.

Statistics

For all statistical analyses, confirm that the following items are present in the figure legend, table legend, main text, or Methods section.

n/a	Confirmed	
☐	☒	The exact sample size (*n*) for each experimental group/condition, given as a discrete number and unit of measurement
☐	☒	A statement on whether measurements were taken from distinct samples or whether the same sample was measured repeatedly
☐	☒	The statistical test(s) used AND whether they are one- or two-sided<br>*Only common tests should be described solely by name; describe more complex techniques in the Methods section.*
☐	☒	A description of all covariates tested
☐	☒	A description of any assumptions or corrections, such as tests of normality and adjustment for multiple comparisons
☐	☒	A full description of the statistical parameters including central tendency (e.g. means) or other basic estimates (e.g. regression coefficient) AND variation (e.g. standard deviation) or associated estimates of uncertainty (e.g. confidence intervals)
☐	☒	For null hypothesis testing, the test statistic (e.g. *F*, *t*, *r*) with confidence intervals, effect sizes, degrees of freedom and *P* value noted<br>*Give P values as exact values whenever suitable.*
☒	☐	For Bayesian analysis, information on the choice of priors and Markov chain Monte Carlo settings
☒	☐	For hierarchical and complex designs, identification of the appropriate level for tests and full reporting of outcomes
☐	☒	Estimates of effect sizes (e.g. Cohen's *d*, Pearson's *r*), indicating how they were calculated

Our web collection on statistics for biologists contains articles on many of the points above.

Software and code

Policy information about availability of computer code

Data collection	Single cell data were acquired using FLow cytometery index sorting and performing the Smart-seq2 protocol for the generation of scRNAseq and index sorting protein expression data. Sequences were generated with Illumina MiSeq or Nextseq machines. Flow cytometry data were acquired using FlowJo 10.4.2 software (FlowJo, LLC).

Data analysis	Statistical analyses were conducted using either Rstudio software 2022.02.1, R 4.1.2, or GraphPad Prism 7.0 (GraphPad Software, Inc., La Jolla, USA) .<br>R package: R package seurat (version 3) was used for integration, unsupervised clustering, differential gene and protein analysis. ggplot2 (3.3.5) was used for data visualization. slingshot (2.2.0) and scanpy (v1.7.1) were used to calculate pseudotime values. clusterProfiler (4.2.2) was used for GSEA analysis. UMAP (0.2.7.0) was used to generate the UMAP coordinates.<br>Scanpy (v1.7.1) was used to perform PAGA and trajectory analysis.<br>The Immunarch R package (v0.6.5) was used for TCR analysis.<br>Trim_Galore (version 0.4.5_dev), BWA-MEM v0.7.17 9 and the Bioconductor packages ATACseqQC package v1.10.1 10 and csaw (v1.20.0) were used for ATAC-seq data analysis.<br><br>The script files to perform all analysis to reproduce the data/results in the paper as well as recreate all figures are deposited at Zenodo, with the following link:<br>https://zenodo.org/badge/latestdoi/364361401https://doi.org/10.5281/zenodo.6473570.

For manuscripts utilizing custom algorithms or software that are central to the research but not yet described in published literature, software must be made available to editors and reviewers. We strongly encourage code deposition in a community repository (e.g. GitHub). See the Nature Portfolio guidelines for submitting code & software for further information.

Data

Policy information about availability of data

All manuscripts must include a data availability statement. This statement should provide the following information, where applicable:

- Accession codes, unique identifiers, or web links for publicly available datasets
- A description of any restrictions on data availability
- For clinical datasets or third party data, please ensure that the statement adheres to our policy

Data availability
Single cell RNA seq data are available on Accession number GSE196330 [https://www.ncbi.nlm.nih.gov/geo/query/acc.cgi?acc=GSE196330]. All the single cell RNA-seq data are deposited with GSE196330. All the other data generated and analysed during this study including ATAC-seq, Flow cytometry data as well as viral deep sequencing are all available upon request to the authors.

Human research participants

Policy information about studies involving human research participants and Sex and Gender in Research.

Reporting on sex and gender	Sex was recorded from the participant of the original study cohort. Sex was determined based on self-reporting. Sex was not considered in the study design, which was based on prospectively following people who inject drugs with high risk of infection with hepatitis C virus. Sex has been reported in Supplementary Table 1.
Population characteristics	Subjects were selected from the Hepatitis C Incidence and Transmission Studies (HITS) in prisons and community (HITS-p, HITS-c) cohorts, which prospectively recruited high-risk injecting drug users from New South Wales, Australia.<br>Participants were 18 years or older, with a mean age of 28. Approximatley 60% of the participants were male. More details of the demographics and risk behavior of the participants can be found on the published work as described in the Methods.<br>Eligible participants had a lifetime history of injecting drug use and were documented to be anti-HCV and RNA-HCV negative in the 12 months prior to enrolment. Following initial detection of viremia, blood samples were collected frequently over a 24-week period until spontaneous clearance or chronic infection was established. HCV antibody (Ab) and HCV RNA testing was performed as previously described.
Recruitment	Eligible participants had a lifetime history of injecting drug use and were documented to be anti-HCV and RNA-HCV negative in the 12 months prior to enrolment.<br>Participants were enrolled into the HITS-p cohort if they: were currently incarcerated in a NSW prison; had reported a life-time history of injecting drug use (enrolled 2005–2009) or a life-time history of any risk factors for blood-borne virus transmission (i.e., injecting drug use, tattooing, piercing, blood fights; enrolled 2012–2014); and had negative anti-HCV antibody status documented prior to recruitment.<br>Recruitment for the HITS-p cohort was by posters and word of mouth In both cohorts those recruited were comparable in age, gender and risk behaviour to the people who inject drugs (PWID) populations in the relevant setting (prisons or community). In both cohorts, following detection of new onset viremia, participants were offered enrolment into the more intensive follow-up substudy for incident cases.<br>The findings of this study pertain solely to the understanding of the immune response in early phase of primary HCV infection in humans, which is unlikely to be affected by recruitment of participants.
Ethics oversight	Human research ethics approvals were obtained from Human Research Ethics Committees from the University of New South Wales Human Research Ethics Committee (HC190074). Written informed consent was obtained from the participants. All methods were performed in accordance with the relevant guidelines and regulations.

Note that full information on the approval of the study protocol must also be provided in the manuscript.

Field-specific reporting

Please select the one below that is the best fit for your research. If you are not sure, read the appropriate sections before making your selection.

- [x] Life sciences
- [ ] Behavioural & social sciences
- [ ] Ecological, evolutionary & environmental sciences

For a reference copy of the document with all sections, see nature.com/documents/nr-reporting-summary-flat.pdf

Life sciences study design

All studies must disclose on these points even when the disclosure is negative.

Sample size	Subjects for this study were chosen based on availability of longitudinal blood samples from a prospective cohort of subjects which were followed from pre-infection, through the primary infections, and up to 2 years post-infection. The selection criteria included available viral sequencing, to identify autologous viral epitopes.
Data exclusions	No Data were excluded.
Replication	IFN-gamma ELISPOT data obtained using a matrix-based approach were multiple epitopes are tested simultaneously and each epitope is tested at least two times. Positive responses were validated in a second experiment the day after the first test with specific peptides , as explained in the method. The details of this approach is outlined in the references reported in the Supplementary note.
Randomization	No randomisation was performed. Subjects were analysed by infection outcome. Given the limited sample size no multi-variate analysis was performed.
Blinding	Not applicable for this study. Individuals were recruited on a prospective cohort and followed longitudinally. Recruitment was based on negative HCV antibody and RNA virus tests. The longitudinal follow up was based on monitoring infection cases without stratification.

Reporting for specific materials, systems and methods

We require information from authors about some types of materials, experimental systems and methods used in many studies. Here, indicate whether each material, system or method listed is relevant to your study. If you are not sure if a list item applies to your research, read the appropriate section before selecting a response.

Materials & experimental systems

n/a	Involved in the study	
[ ]	[x]	Antibodies
[x]	[ ]	Eukaryotic cell lines
[x]	[ ]	Palaeontology and archaeology
[x]	[ ]	Animals and other organisms
[x]	[ ]	Clinical data
[x]	[ ]	Dual use research of concern

Methods

n/a	Involved in the study	
[x]	[ ]	ChIP-seq
[ ]	[x]	Flow cytometry
[x]	[ ]	MRI-based neuroimaging

Antibodies

Antibodies used

Antibodies used for this study are listed below. Details on staining are provided in the supplementary Note.
Two panels of antibodies were used for flow cytometry analysis. Both panels contained FITC anti-CD4 (RPA-T4), PE-Cy5 anti-CD19 (HIB19), APC-Cy7 anti-CD3 (SK7), AF700 anti-CD8 (RPA-T8), BV510 anti-PD-1 (EH12.1). The first panel included: BV421 and PerCP/Cy5.5 anti-TIM-3 (RMT3-23, BioLegend), BV605 anti-CD38 (HB7), BV650 anti-CD127 (HIL-7R-M21), PE-Vio770 anti-2B4 (REA112, Miltenyi Biotec), AF647 anti-CD160 (BY55, BioLegend), BV650 CD127 (HIL-7R-M21). Dextramers (Immudex) were conjugated with PE. For KLRG1 staining, cells were incubated with primary antibody Biotin anti-KLRG1 (2F1) followed by incubation with secondary antibody PE-CF594 Streptavidin.

The second panel included BV421 CCR7 (150503), PE-Cy-7 CD45RO (UCHL1), BUV395 CD27 (L128), PE-CF594 CTLA-4 (BNI3). For the intracellular staining, cells were fixed and permeabilized using fix/perm buffer from transcription factor buffer set kit (BD Biosciences) at 4°C for 35 minutes and stained with against intracellular markers anti T-bet BV711 (clone O4-46), anti EOMES eFluor660 (WD1928, eBioscience) at 4°C for 30 minutes. The cells were then washed twice with Perm/Wash buffer (BD Biosciences) and fixed with PBS containing 1% paraformaldehyde.

Fluorophore-conjugated antibodies used in this study.

Target	Fluorophore	Clone	Vendor	Catalogue number	Dilution
CD4	FITC	RPA-T4	BD	555346	1:20
CD19	PE-Cy5	HIB19	BD	555414	1:20

CD3	APC-Cy7	SK7	BD	557832	1:10
CD8	AF700	RPA-T8	BD	561026	1:10
PD-1	BV510	EH12.1	BD	563076	1:20
TIM-3	BV421	F38-2E2	BioLegend	345008	1:20
TIM-3	PerCP-Cy5.5	F38-2E2	BioLegend	345016	1:20
CD38	BV605	HB7	BD	562665	1:20
CD127	BV650	HIL-7R-M21	BD	563225	1:20
2B4	PE-Vio770	REA112	Miltenyi Biotec	130-099-074	1:20
CD160	AF647	BY55	BioLegend	341204	1:20
Dextramer	PE	Various	Immudex	Various	1:20
KLRG1	Biotin	2F1	Biolegend	138406	1:100
Streptavidin	PE-CF594	None	BD	562284	1 :100
CCR7	BV421	150503	BD	562555	1:20
CD45RO	PE-Cy-7	UCHL1	BD	337168	1:20
CD27	BUV395	L128	BD	563816	1:20
CTLA-4	PE-CF594	BNI3	BD	562742	1:20
T-bet	BV711	O4-46	BD	563320	1:20
EOMES	eFluor660	WD1928	eBioscience	50-4877-42	1:20
CD3	BV480	UCHT1	BD	566105	1:50
CD122	BV650	MiK-β3	BD	743117	1:40
CD95	BV786	DX2	BD	740991	1:80
CD38	APC	HB7	BD	345807	1:20
CD8	APC-R700	RPA-T8	BD	565165	1:10
PD-1	PE-CF594	EH12.1	BD	565024	1:20
CD127	PE-Cy7	HIL-7R-M21	BD	560822	1:10
CD45RA	FITC	HI100	BD	555488	1:5
KLRG1	PerCP-Cy5.5	SA231A2	BioLegend	367708	1:20
CXCR3	APC	1C6/CXCR3	BD	550967	1:20

Validation

Antibodies were all obtained from commercial manufacturers and were quality controlled and tested as part of routine analyses and as recommended by the manufacturer's web site.

Flow Cytometry

Plots

Confirm that:

☒ The axis labels state the marker and fluorochrome used (e.g. CD4-FITC).

☒ The axis scales are clearly visible. Include numbers along axes only for bottom left plot of group (a 'group' is an analysis of identical markers).

☒ All plots are contour plots with outliers or pseudocolor plots.

☒ A numerical value for number of cells or percentage (with statistics) is provided.

Methodology

Sample preparation

Cryopreserved PBMC were used for this study. For flow cytomtry, Peripheral blood mononuclear cells (PBMCs) were thawed in RPMI and washed with PBS containing 1% BSA. Cells were stained with PE-conjugated HCV-specific class I dextramers (Immudex, Copenhagen, Denmark) at room temperature, followed by viability staining (LIVE/DEADTM fixable blue for analysis or fixable yellow for sorting) (Invitrogen, Carlsbad, CA) and staining with panels of surface or intracellular antibodies detailed below.

Instrument

Flow cytometry was performed using the LSR Fortessa analyser, and FACSAria III and Influx sorters (BD Biosciences, San Diego, CA).

Software

Flow cytometry data was analysed using FlowJo version 10.1 (BD Biosciences, San Diego, CA).

Cell population abundance

Target populations were sorted with single-cell precision.

Gating strategy

A gating strategy has been provided in supplementary figure 2. Lymphocytes were selected by forward and side scatter. Single cells were gated and non viable and CD19+ cells were excluded. The target population of HCV-specific CD8+ T cells for sorting was identified using dextramers. Gates for CD127, PD-1, CD38, and Tim-3 were determined with fluorescence minus one (FMO) controls.

☒ Tick this box to confirm that a figure exemplifying the gating strategy is provided in the Supplementary Information.
